# Supplementary material for: Mapping the evolution of fertility support policies in China: A content and instrumental analysis
Source: PLoS One. 2025 Oct 9;20(10):e0332137. doi: 10.1371/journal.pone.0332137 (PMC12510515; doi:10.1371/journal.pone.0332137)
Supplement: S1 Appendix — (ZIP) [file pone.0332137.s001.zip › S1 Appendix. 226 original policy documents/222-财政部、税务总局、发展改革委等关于养老、托育、家政等社区家庭服务业税费优惠政策的公告(FBM-CLI-4-333770).doc]

财政部、税务总局、发展改革委等关于养老、托育、家政等社区家庭服务业税费优惠政策的公告

**发布部门：** [财政部](https://www.pkulaw.com/law/chl?Aggs.IssueDepartment=60210) [国家税务总局](https://www.pkulaw.com/law/chl?Aggs.IssueDepartment=60401) [国家发展和改革委员会(含原国家发展计划委员会、原国家计划委员会)](https://www.pkulaw.com/law/chl?Aggs.IssueDepartment=60301) [民政部](https://www.pkulaw.com/law/chl?Aggs.IssueDepartment=60208) [商务部](https://www.pkulaw.com/law/chl?Aggs.IssueDepartment=60220) [国家卫生健康委员会](https://www.pkulaw.com/law/chl?Aggs.IssueDepartment=60321) [机构沿革](javascript:void(0))

**发文字号：**财政部公告2019年第76号

**发布日期：**2019.06.28

**实施日期：**2019.06.01

**时效性：** [现行有效](https://www.pkulaw.com/law/chl?Aggs.TimelinessDic=01)

**效力级别：** [部门规范性文件](https://www.pkulaw.com/law/chl?Aggs.EffectivenessDic=XE0302)

**法规类别：** [税收优惠](https://www.pkulaw.com/law/chl?Aggs.Category=0320504) [税收综合规定](https://www.pkulaw.com/law/chl?Aggs.Category=03202) [营商环境优化](https://dobusiness.pkulaw.com/)

财政部、税务总局、发展改革委、民政部、商务部、卫生健康委关于养老、托育、家政等社区家庭服务业税费优惠政策的公告
（财政部公告2019年第76号）

　　为支持养老、托育、家政等社区家庭服务业发展，现就有关税费政策公告如下：
　　**一、**为社区提供养老、托育、家政等服务的机构，按照以下规定享受税费优惠政策：
　　（一）提供社区养老、托育、家政服务取得的收入，免征增值税。
　　（二）提供社区养老、托育、家政服务取得的收入，在计算应纳税所得额时，减按90%计入收入总额。
　　（三）承受房屋、土地用于提供社区养老、托育、家政服务的，免征契税。
　　（四）用于提供社区养老、托育、家政服务的房产、土地，免征不动产登记费、耕地开垦费、土地复垦费、土地闲置费；用于提供社区养老、托育、家政服务的建设项目，免征城市基础设施配套费；确因地质条件等原因无法修建防空地下室的，免征防空地下室易地建设费。

　　**二、**为社区提供养老、托育、家政等服务的机构自有或其通过承租、无偿使用等方式取得并用于提供社区养老、托育、家政服务的房产、土地，免征房产税、城镇土地使用税。

　　**三、**本公告所称社区是指聚居在一定地域范围内的人们所组成的社会生活共同体，包括城市社区和农村社区。
　　为社区提供养老服务的机构，是指在社区依托固定场所设施，采取全托、日托、上门等方式，为社区居民提供养老服务的企业、事业单位和社会组织。社区养老服务是指为老年人提供的生活照料、康复护理、助餐助行、紧急救援、精神慰藉等服务。
　　为社区提供托育服务的机构，是指在社区依托固定场所设施，采取全日托、半日托、计时托、临时托等方式，为社区居民提供托育服务的企业、事业单位和社会组织。社区托育服务是指为3周岁（含）以下婴幼儿提供的照料、看护、膳食、保育等服务。
　　为社区提供家政服务的机构，是指以家庭为服务对象，为社区居民提供家政服务的企业、事业单位和社会组织。社区家政服务是指进入家庭成员住所或医疗机构为孕产妇、婴幼儿、老人、病人、残疾人提供的照护服务，以及进入家庭成员住所提供的保洁、烹饪等服务。

　　**四、**符合下列条件的家政服务企业提供家政服务取得的收入，比照《营业税改征增值税试点过渡政策的规定》（财税〔2016〕36号附件）第一条第（三十一）项规定，免征增值税。
　　（一）与家政服务员、接受家政服务的客户就提供家政服务行为签订三方协议；
　　（二）向家政服务员发放劳动报酬，并对家政服务员进行培训管理；
　　（三）通过建立业务管理系统对家政服务员进行登记管理。

　　**五、**财政、税费征收机关可根据工作需要与民政、卫生健康、商务等部门建立信息共享和工作配合机制，民政、卫生健康、商务等部门应积极协同配合，保障优惠政策落实到位。

　　**六、**本公告自2019年6月1日起执行至2025年12月31日。

财政部
税务总局
发展改革委
民政部
商务部
卫生健康委
2019年6月28日

© 北大法宝：（[www.pkulaw.com](https://www.pkulaw.com/)) 专业提供法律信息、法学知识和法律软件领域各类解决方案。北大法宝为您提供丰富的参考资料，正式引用法规条文时请与标准文本核对。
欢迎查看所有[产品和服务](https://www.pkulaw.com/helps/2.html)。法宝快讯：[如何快速找到您需要的检索结果？](https://www.pkulaw.com/helps/70.html)[法宝](https://www.pkulaw.com/helps/69.html)V6有何新特色？

扫描二维码阅读原文

**原文链接：**<https://www.pkulaw.com/chl/b6d0439b33cea95abdfb.html>
